# Supplementary figures and images for: Comparative genomics of two inbred lines of the potato cyst nematode Globodera rostochiensis reveals disparate effector family-specific diversification patterns
Source: BMC Genomics. 2021 Aug 11;22:611. doi: 10.1186/s12864-021-07914-6 (PMC8359618; doi:10.1186/s12864-021-07914-6)

## Slide 1
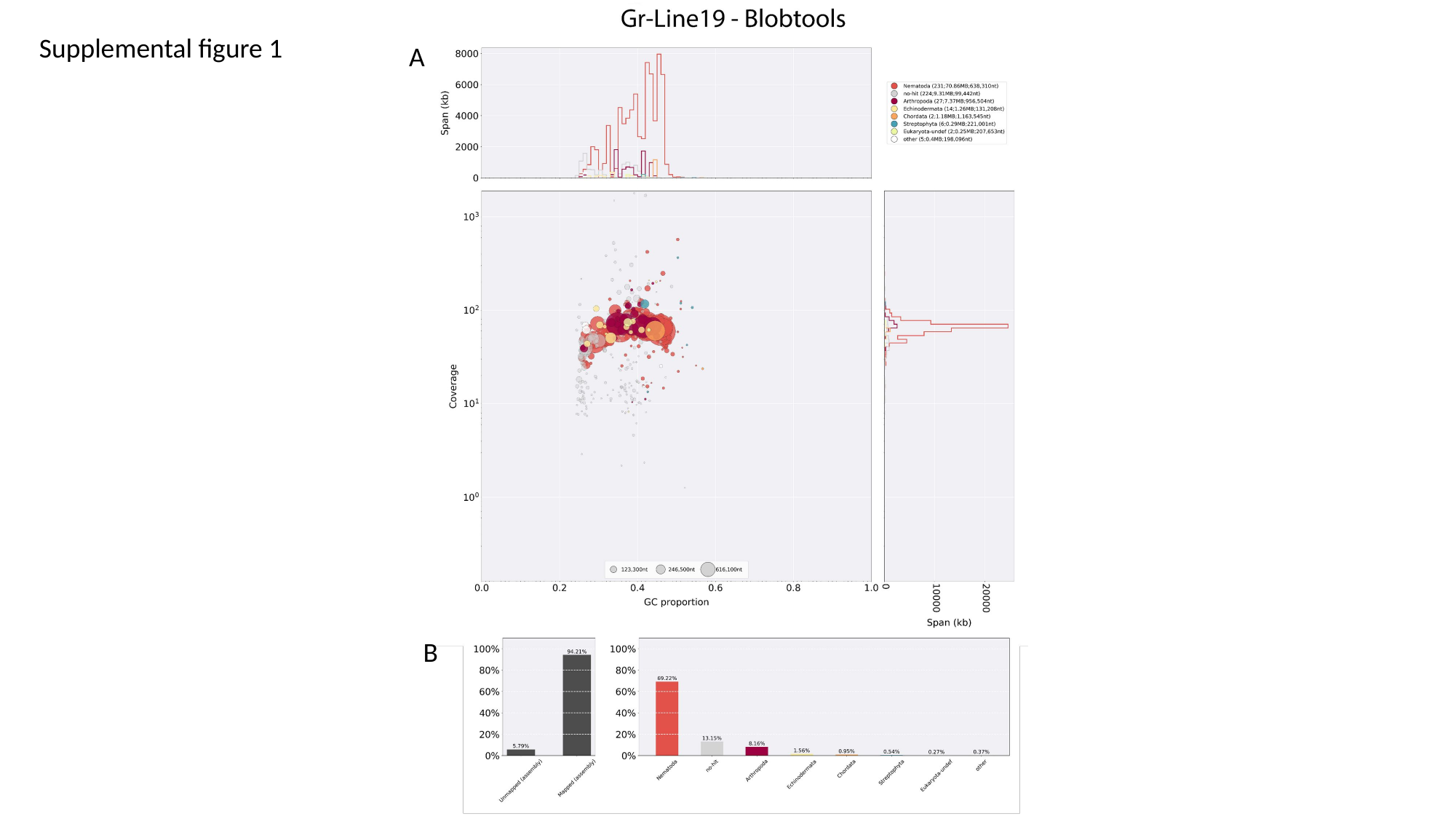

Supplemental figure 1
A
B

## Slide 2
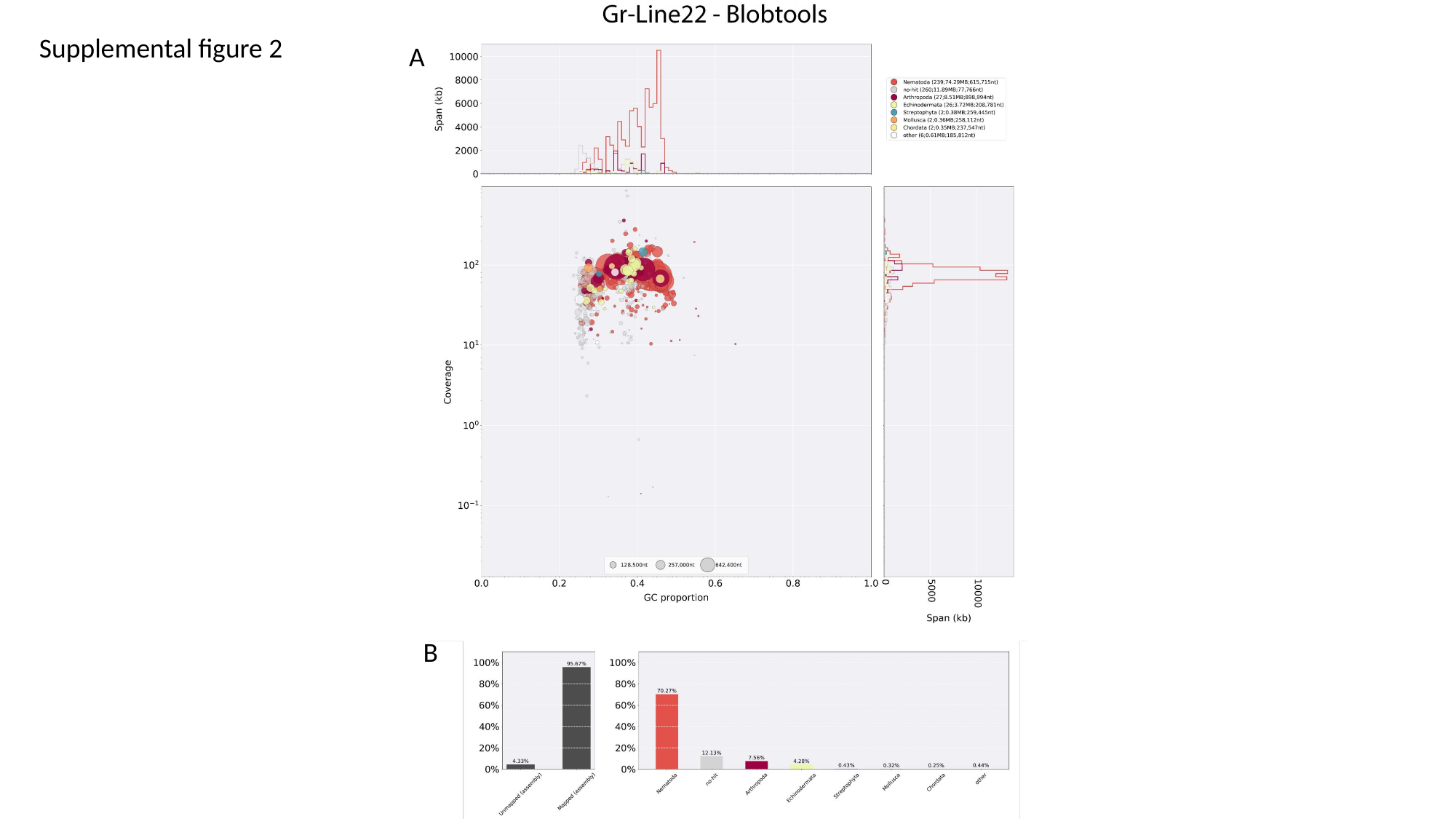

Supplemental figure 2
A
B

## Slide 3
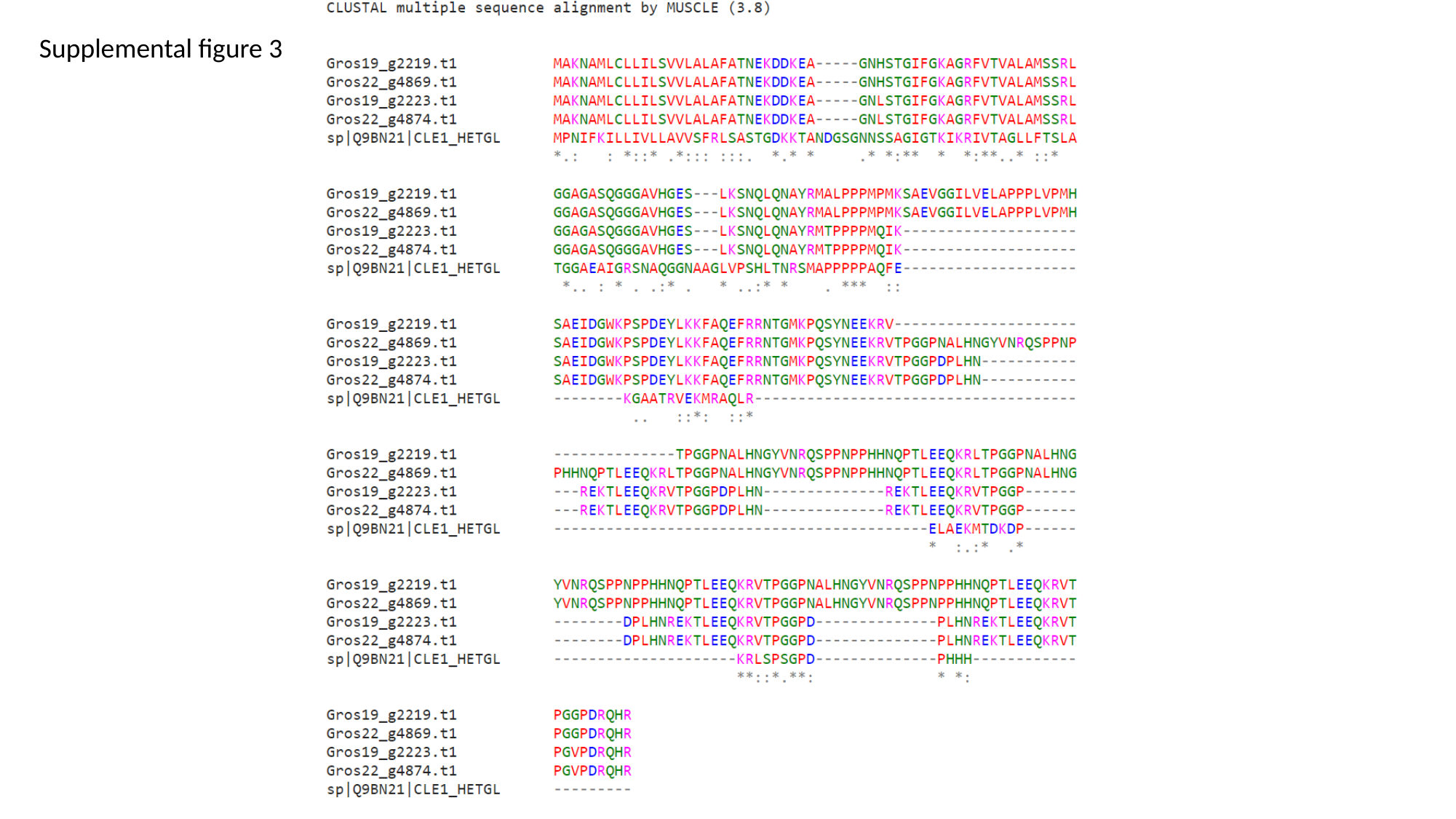

Supplemental figure 3

Supplement: Supplementary file 1 — Additional file 1: Figure S1 - BlobTools-based interrogation of genome assembly of Gr-Line 19 to verify for single-taxon origin of the original sequences. Panel A: Each Gr-Line19 scaffold is represented by a single filled circle. Each scaffold is positioned in the main panel based on its GC proportion (x-axis) and coverage by reads from PacBio sequences (y-axis). On the top right the colours of the individual blobs are linked to their taxonomic origin. At the bottom of the main Blobtool figure, the size of the circles is linked to scaffold size. Panel B: on the left the % of unmapped versus mapped Gr-Line19 PacBio reads are presented, and the right the taxonomic origin of the reads. Figure S2 - BlobTools-based interrogation of genome assembly of Gr-Line 22 to verify for single-taxon origin of the original sequences. Panel A: Each Gr-Line22 scaffold is represented by a single filled circle. Each scaffold is positioned in the main panel based on its GC proportion (x-axis) and coverage by reads from PacBio sequences (y-axis). On the top right the colours of the individual blobs are linked to their taxonomic origin. At the bottom of the main Blobtool figure, the size of the circles is linked to scaffold size. Panel B: on the left the % of unmapped versus mapped Gr-Line22 PacBio reads are presented, and the right the taxonomic origin of the reads. Figure S3 - Multiple sequence alignment of Gr-CLE-1 protein sequences to verify the conservation of the CLE domain in putative CLE-1 genes. Each gene is represented by a gene identifier. Two genes are included for Gr-Line19 (Gros19_g2219.t1 and Gros19_g2223.t1) and two for Gr-Line22 (Gros22_g4869.t1 and Gros22_g4874.t1). The CLE1 sequence identified in Heterodera glycines (Q9BN21) is included as an outgroup. [file 12864_2021_7914_MOESM1_ESM.pptx]
